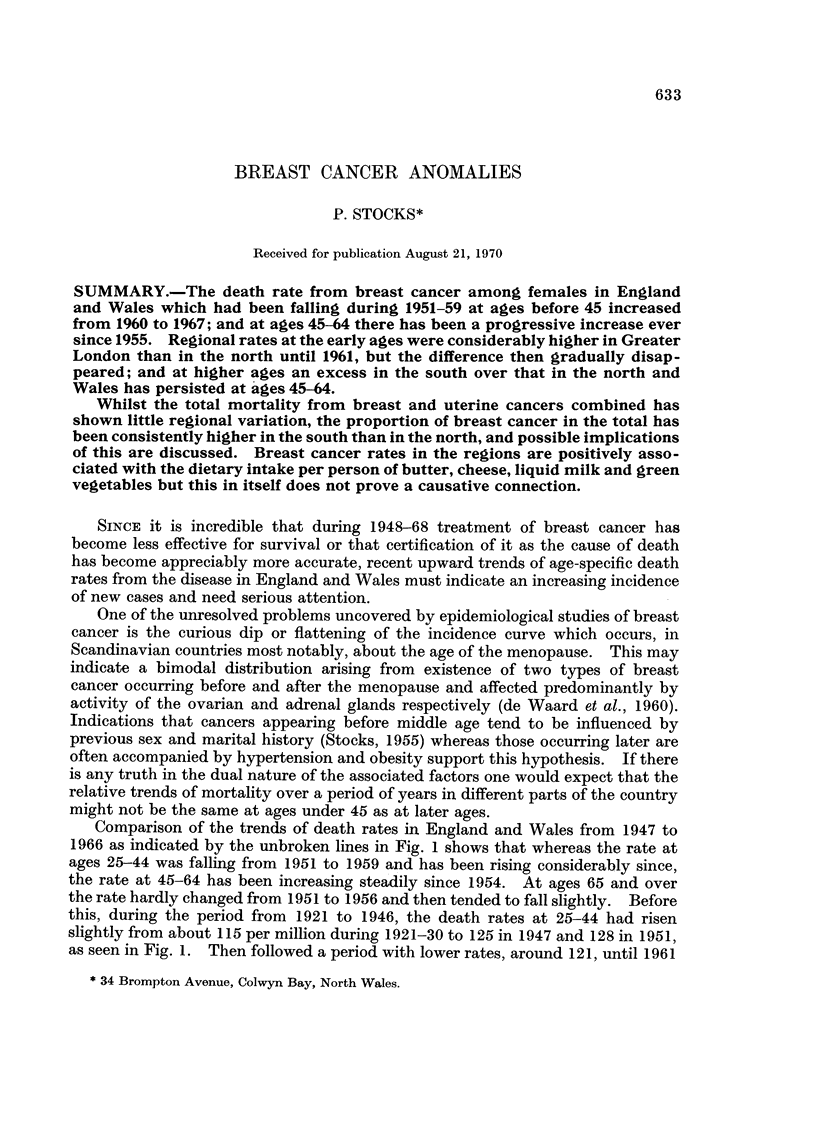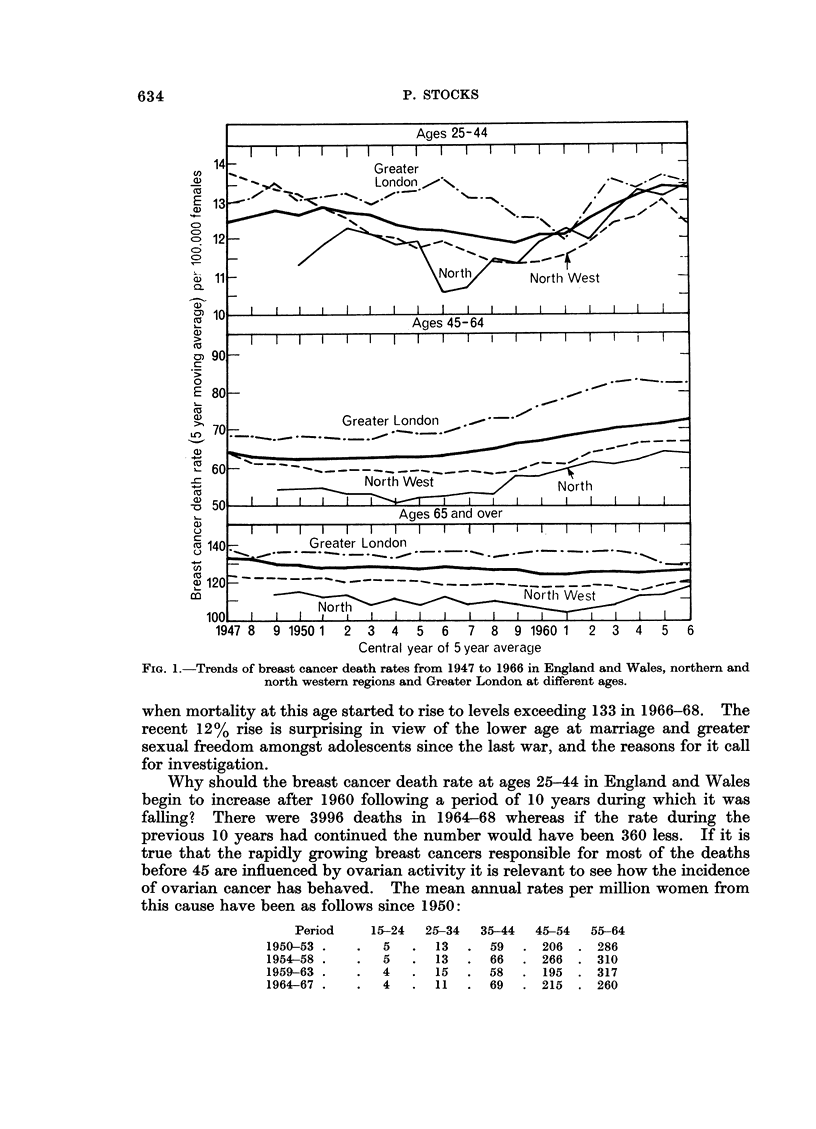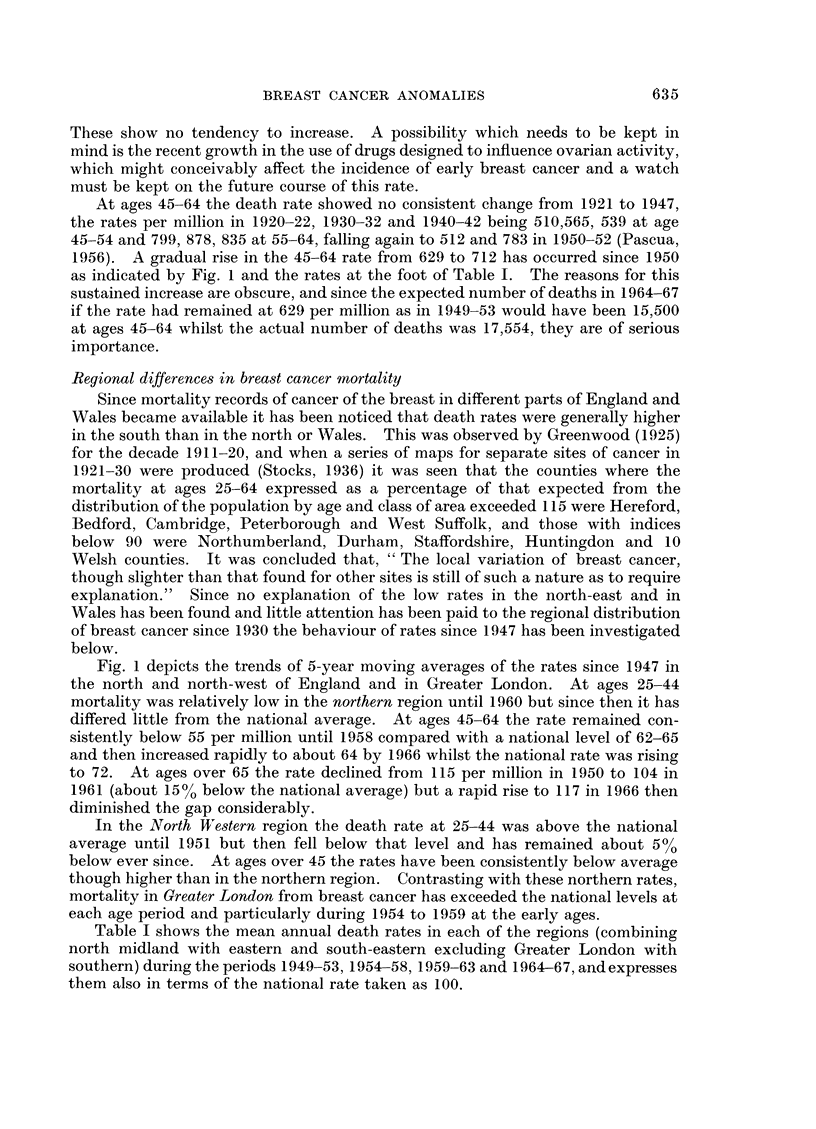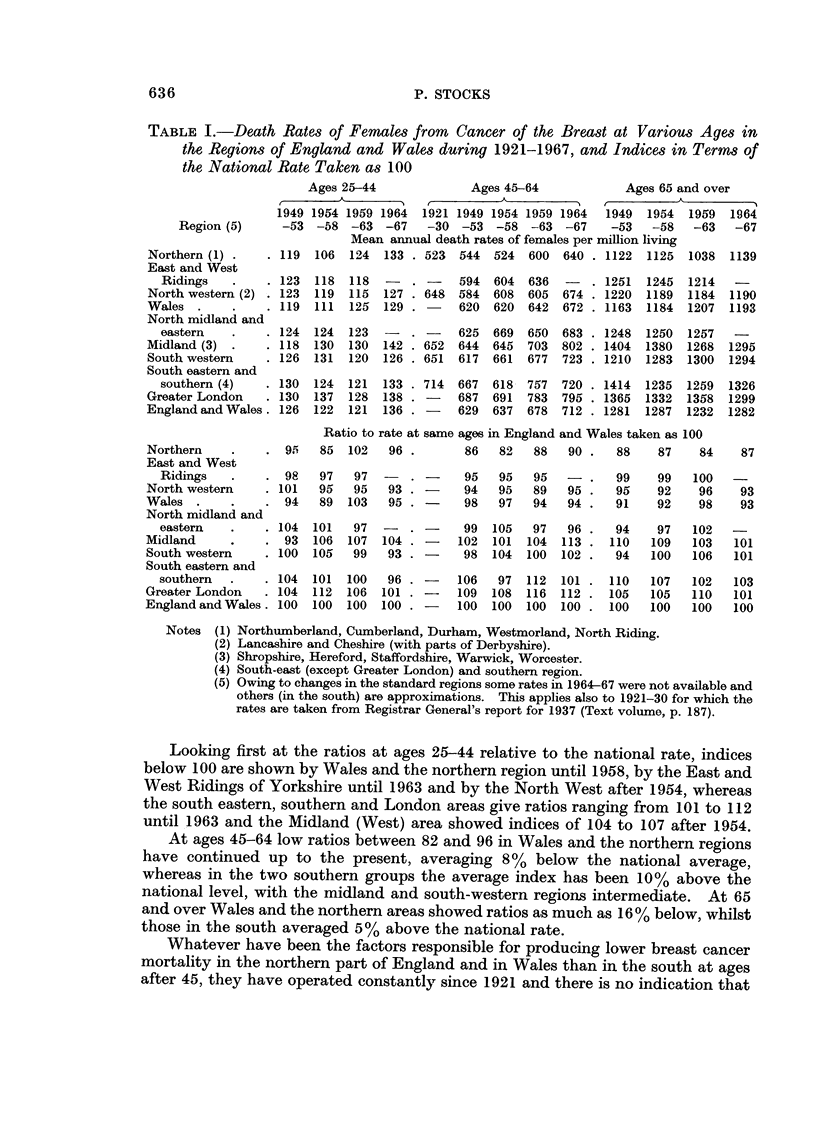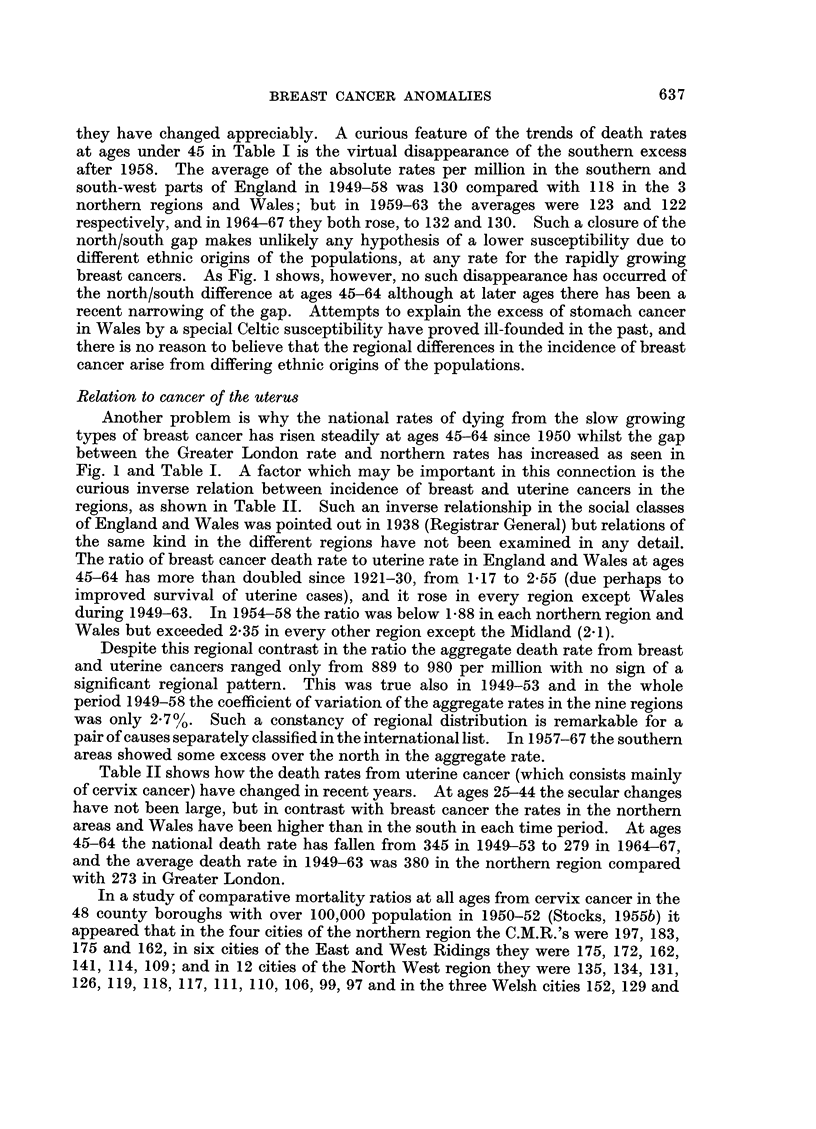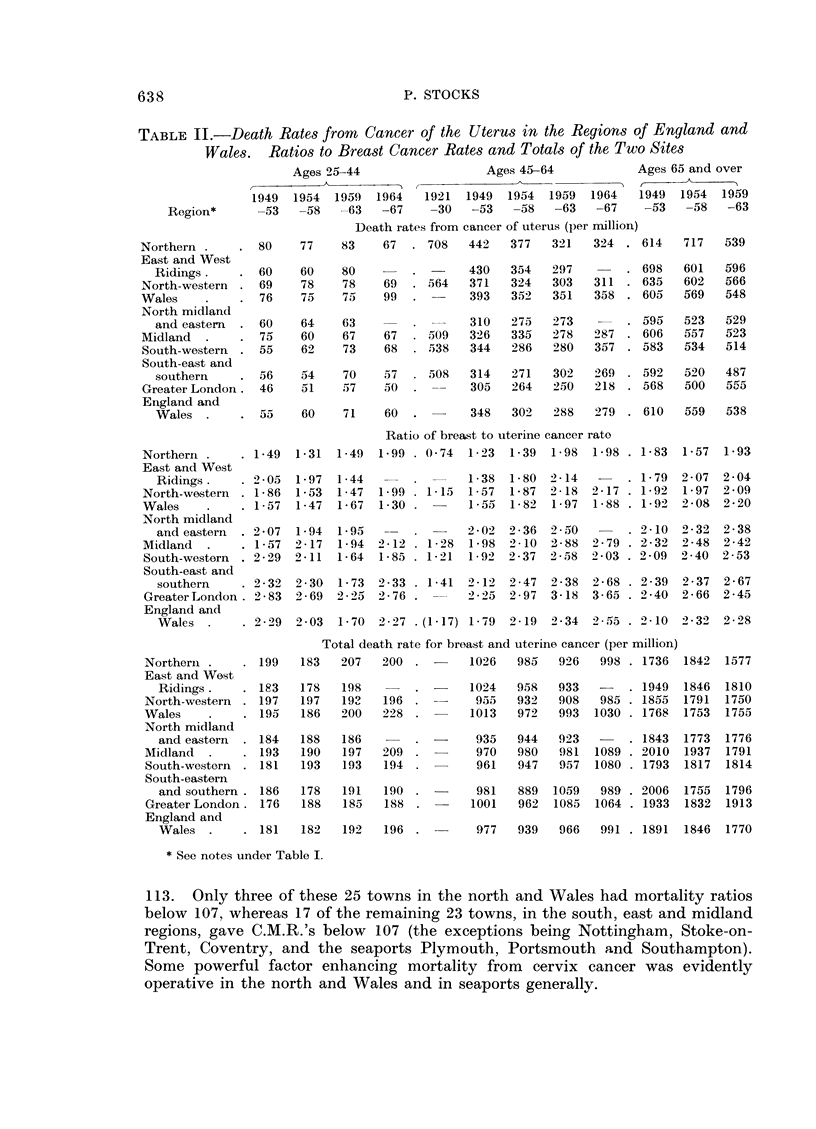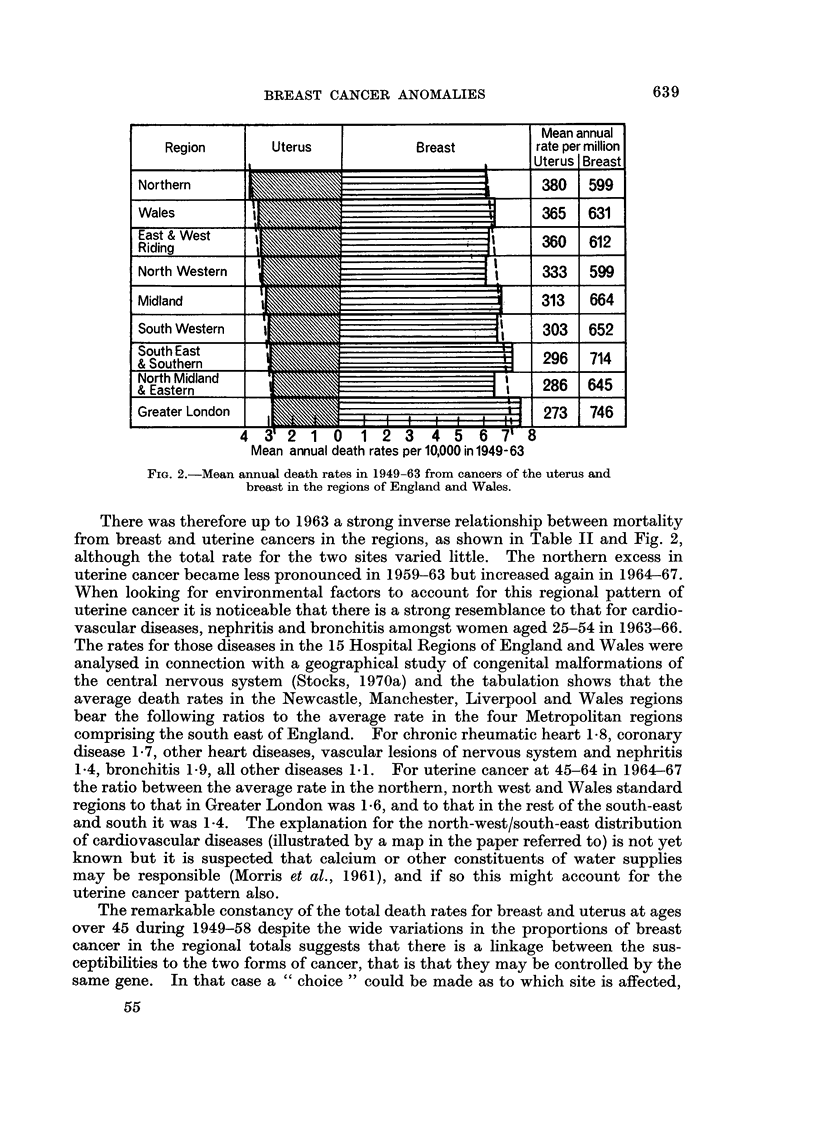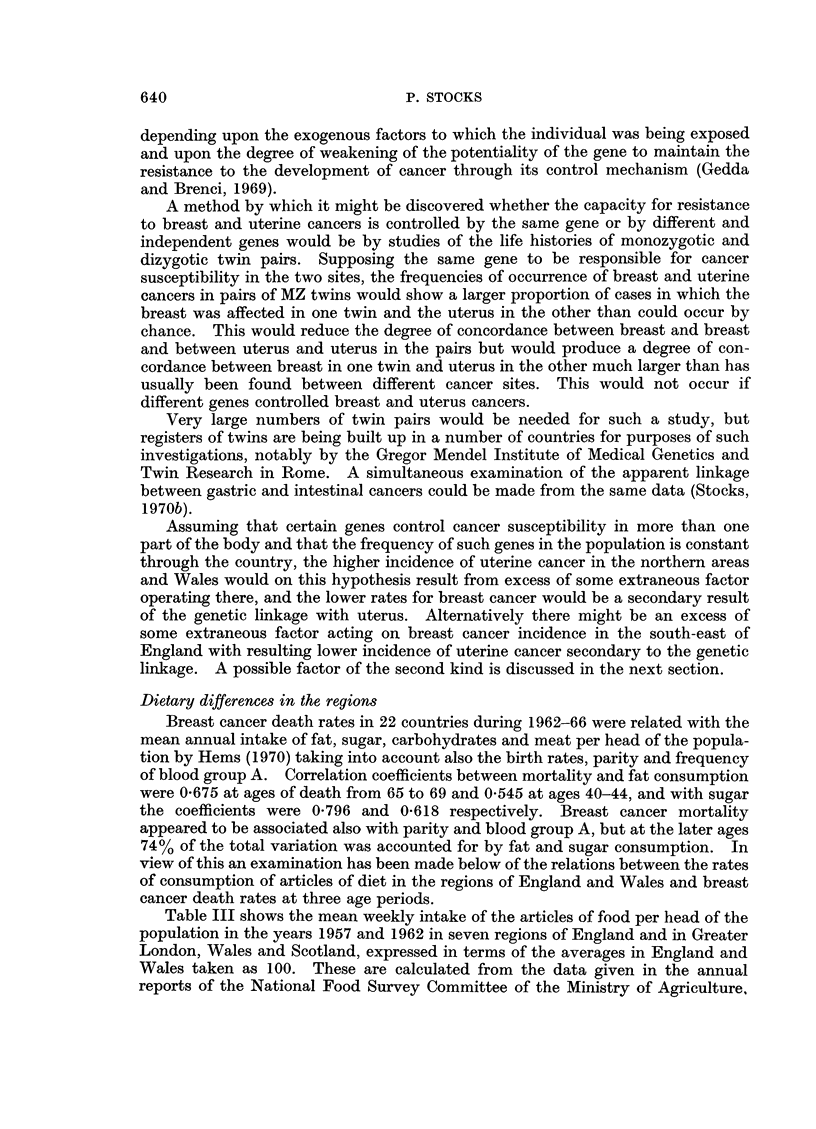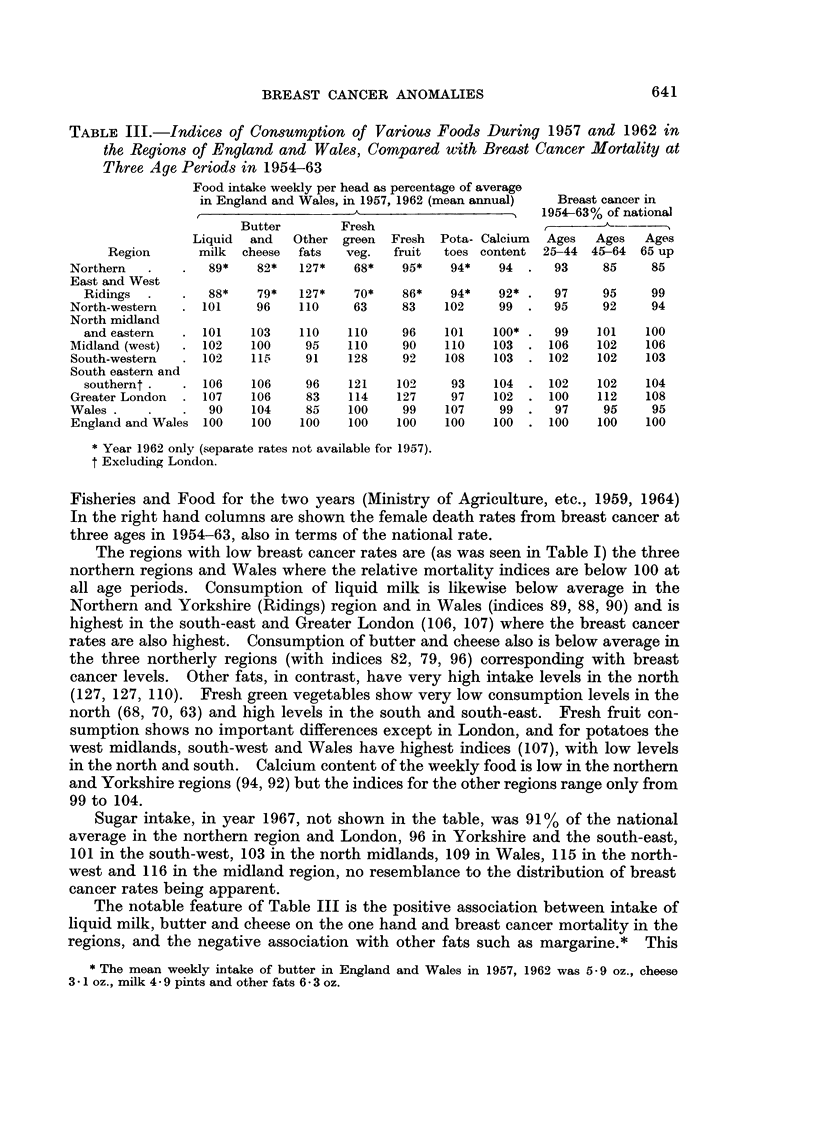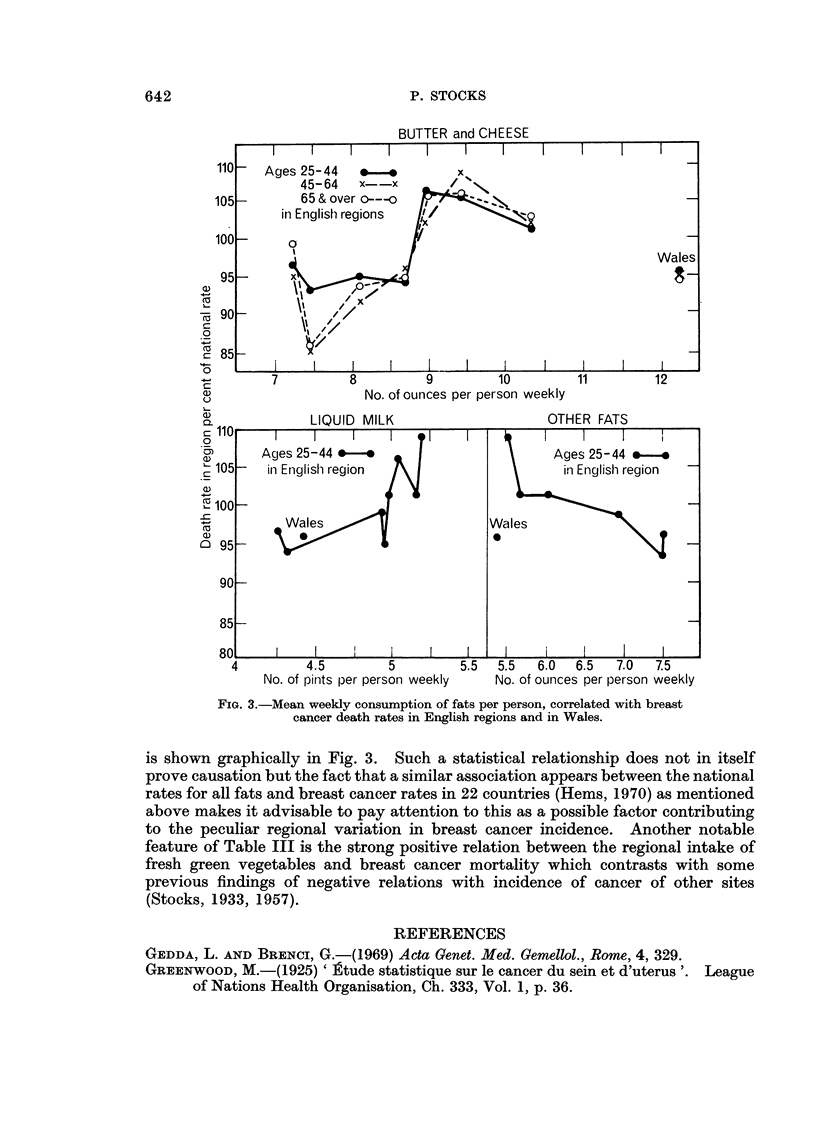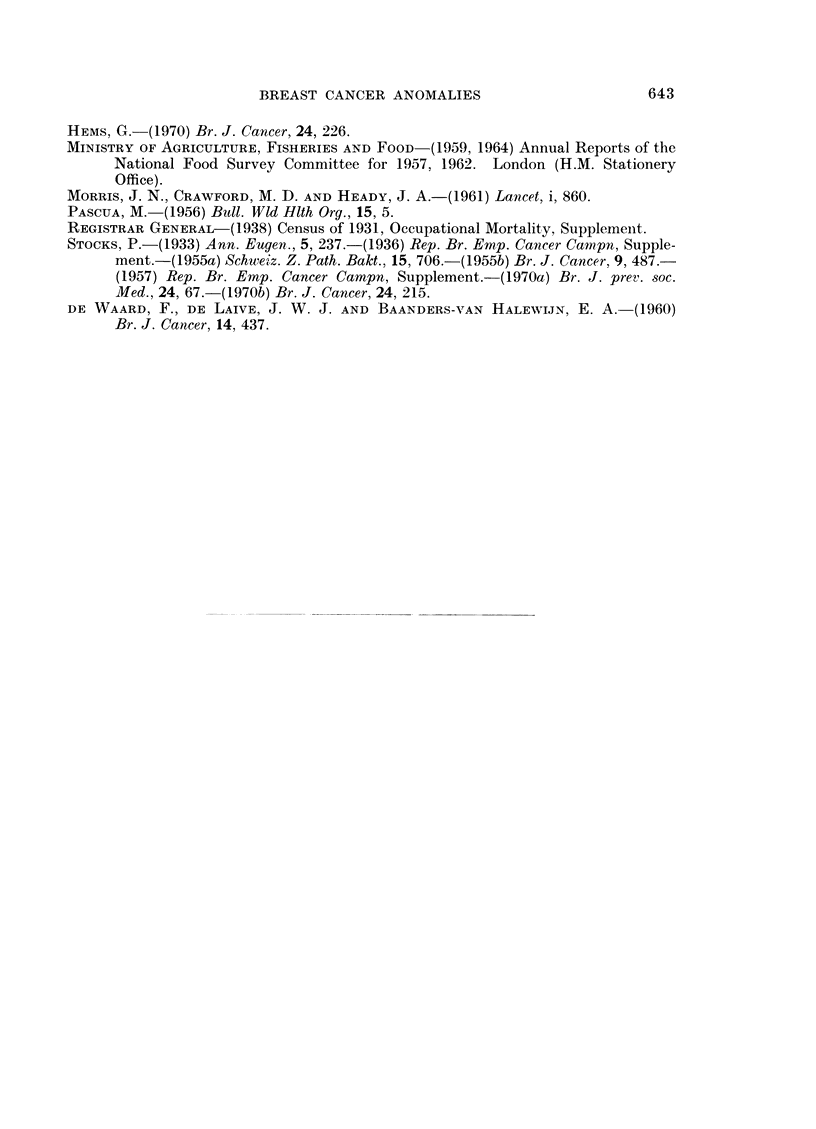# Breast Cancer Anomalies

**DOI:** 10.1038/bjc.1970.77

**Published:** 1970-12

**Authors:** P. Stocks

## Abstract

The death rate from breast cancer among females in England and Wales which had been falling during 1951-59 at ages before 45 increased from 1960 to 1967; and at ages 45-64 there has been a progressive increase ever since 1955. Regional rates at the early ages were considerably higher in Greater London than in the north until 1961, but the difference then gradually disappeared; and at higher ages an excess in the south over that in the north and Wales has persisted at ages 45-64.

Whilst the total mortality from breast and uterine cancers combined has shown little regional variation, the proportion of breast cancer in the total has been consistently higher in the south than in the north, and possible implications of this are discussed. Breast cancer rates in the regions are positively associated with the dietary intake per person of butter, cheese, liquid milk and green vegetables but this in itself does not prove a causative connection.


### BREAST CANCER ANOMALIES

P. STOCKS*

Received for publication August 21, 1970

**SUMMARY.—The death rate from breast cancer among females in England and Wales which had been falling during 1951–59 at ages before 45 increased from 1960 to 1967; and at ages 45–64 there has been a progressive increase ever since 1955. Regional rates at the early ages were considerably higher in Greater London than in the north until 1961, but the difference then gradually disappeared; and at higher ages an excess in the south over that in the north and Wales has persisted at ages 45–64.**

**Whilst the total mortality from breast and uterine cancers combined has shown little regional variation, the proportion of breast cancer in the total has been consistently higher in the south than in the north, and possible implications of this are discussed. Breast cancer rates in the regions are positively associated with the dietary intake per person of butter, cheese, liquid milk and green vegetables but this in itself does not prove a causative connection.**

SINCE it is incredible that during 1948–68 treatment of breast cancer has become less effective for survival or that certification of it as the cause of death has become appreciably more accurate, recent upward trends of age-specific death rates from the disease in England and Wales must indicate an increasing incidence of new cases and need serious attention.

One of the unresolved problems uncovered by epidemiological studies of breast cancer is the curious dip or flattening of the incidence curve which occurs, in Scandinavian countries most notably, about the age of the menopause. This may indicate a bimodal distribution arising from existence of two types of breast cancer occurring before and after the menopause and affected predominantly by activity of the ovarian and adrenal glands respectively (de Waard *et al.*, 1960). Indications that cancers appearing before middle age tend to be influenced by previous sex and marital history (Stocks, 1955) whereas those occurring later are often accompanied by hypertension and obesity support this hypothesis. If there is any truth in the dual nature of the associated factors one would expect that the relative trends of mortality over a period of years in different parts of the country might not be the same at ages under 45 as at later ages.

Comparison of the trends of death rates in England and Wales from 1947 to 1966 as indicated by the unbroken lines in Fig. 1 shows that whereas the rate at ages 25–44 was falling from 1951 to 1959 and has been rising considerably since, the rate at 45–64 has been increasing steadily since 1954. At ages 65 and over the rate hardly changed from 1951 to 1956 and then tended to fall slightly. Before this, during the period from 1921 to 1946, the death rates at 25–44 had risen slightly from about 115 per million during 1921–30 to 125 in 1947 and 128 in 1951, as seen in Fig. 1. Then followed a period with lower rates, around 121, until 1961

* 34 Brompton Avenue, Colwyn Bay, North Wales.

FIG. 1.—Trends of breast cancer death rates from 1947 to 1966 in England and Wales, northern and north western regions and Greater London at different ages.

when mortality at this age started to rise to levels exceeding 133 in 1966–68. The recent 12% rise is surprising in view of the lower age at marriage and greater sexual freedom amongst adolescents since the last war, and the reasons for it call for investigation.

Why should the breast cancer death rate at ages 25–44 in England and Wales begin to increase after 1960 following a period of 10 years during which it was falling? There were 3996 deaths in 1964–68 whereas if the rate during the previous 10 years had continued the number would have been 360 less. If it is true that the rapidly growing breast cancers responsible for most of the deaths before 45 are influenced by ovarian activity it is relevant to see how the incidence of ovarian cancer has behaved. The mean annual rates per million women from this cause have been as follows since 1950:

| Period | 15–24 | 25–34 | 35–44 | 45–54 | 55–64 |
|---|---|---|---|---|---|
| 1950–53 | 5 | 13 | 59 | 206 | 286 |
| 1954–58 | 5 | 13 | 66 | 266 | 310 |
| 1959–63 | 4 | 15 | 58 | 195 | 317 |
| 1964–67 | 4 | 11 | 69 | 215 | 260 |

These show no tendency to increase. A possibility which needs to be kept in mind is the recent growth in the use of drugs designed to influence ovarian activity, which might conceivably affect the incidence of early breast cancer and a watch must be kept on the future course of this rate.

At ages 45–64 the death rate showed no consistent change from 1921 to 1947, the rates per million in 1920–22, 1930–32 and 1940–42 being 510,565, 539 at age 45–54 and 799, 878, 835 at 55–64, falling again to 512 and 783 in 1950–52 (Pascua, 1956). A gradual rise in the 45–64 rate from 629 to 712 has occurred since 1950 as indicated by Fig. 1 and the rates at the foot of Table I. The reasons for this sustained increase are obscure, and since the expected number of deaths in 1964–67 if the rate had remained at 629 per million as in 1949–53 would have been 15,500 at ages 45–64 whilst the actual number of deaths was 17,554, they are of serious importance.

*Regional differences in breast cancer mortality*

Since mortality records of cancer of the breast in different parts of England and Wales became available it has been noticed that death rates were generally higher in the south than in the north or Wales. This was observed by Greenwood (1925) for the decade 1911–20, and when a series of maps for separate sites of cancer in 1921–30 were produced (Stocks, 1936) it was seen that the counties where the mortality at ages 25–64 expressed as a percentage of that expected from the distribution of the population by age and class of area exceeded 115 were Hereford, Bedford, Cambridge, Peterborough and West Suffolk, and those with indices below 90 were Northumberland, Durham, Staffordshire, Huntingdon and 10 Welsh counties. It was concluded that, " The local variation of breast cancer, though slighter than that found for other sites is still of such a nature as to require explanation." Since no explanation of the low rates in the north-east and in Wales has been found and little attention has been paid to the regional distribution of breast cancer since 1930 the behaviour of rates since 1947 has been investigated below.

Fig. 1 depicts the trends of 5-year moving averages of the rates since 1947 in the north and north-west of England and in Greater London. At ages 25–44 mortality was relatively low in the *northern* region until 1960 but since then it has differed little from the national average. At ages 45–64 the rate remained consistently below 55 per million until 1958 compared with a national level of 62–65 and then increased rapidly to about 64 by 1966 whilst the national rate was rising to 72. At ages over 65 the rate declined from 115 per million in 1950 to 104 in 1961 (about 15% below the national average) but a rapid rise to 117 in 1966 then diminished the gap considerably.

In the *North Western* region the death rate at 25–44 was above the national average until 1951 but then fell below that level and has remained about 5% below ever since. At ages over 45 the rates have been consistently below average though higher than in the northern region. Contrasting with these northern rates, mortality in *Greater London* from breast cancer has exceeded the national levels at each age period and particularly during 1954 to 1959 at the early ages.

Table I shows the mean annual death rates in each of the regions (combining north midland with eastern and south-eastern excluding Greater London with southern) during the periods 1949–53, 1954–58, 1959–63 and 1964–67, and expresses them also in terms of the national rate taken as 100.

TABLE I.—*Death Rates of Females from Cancer of the Breast at Various Ages in the Regions of England and Wales during* 1921–1967, *and Indices in Terms of the National Rate Taken as* 100

| Region (5) | Ages 25–44 1949–53 | 1954–58 | 1959–63 | 1964–67 | Ages 45–64 1921–30 | 1949–53 | 1954–58 | 1959–63 | 1964–67 | Ages 65 and over 1949–53 | 1954–58 | 1959–63 | 1964–67 |
|---|---|---|---|---|---|---|---|---|---|---|---|---|---|
| | | | | | *Mean annual death rates of females per million living* | | | | | | | | |
| Northern (1) | 119 | 106 | 124 | 133 | 523 | 544 | 524 | 600 | 640 | 1122 | 1125 | 1038 | 1139 |
| East and West Ridings | 123 | 118 | 118 | — | — | 594 | 604 | 636 | — | 1251 | 1245 | 1214 | — |
| North western (2) | 123 | 119 | 115 | 127 | 648 | 584 | 608 | 605 | 674 | 1220 | 1189 | 1184 | 1190 |
| Wales | 119 | 111 | 125 | 129 | — | 620 | 620 | 642 | 672 | 1163 | 1184 | 1207 | 1193 |
| North midland and eastern | 124 | 124 | 123 | — | — | 625 | 669 | 650 | 683 | 1248 | 1250 | 1257 | — |
| Midland (3) | 118 | 130 | 130 | 142 | 652 | 644 | 645 | 703 | 802 | 1404 | 1380 | 1268 | 1295 |
| South western | 126 | 131 | 120 | 126 | 651 | 617 | 661 | 677 | 723 | 1210 | 1283 | 1300 | 1294 |
| South eastern and southern (4) | 130 | 124 | 121 | 133 | 714 | 667 | 618 | 757 | 720 | 1414 | 1235 | 1259 | 1326 |
| Greater London | 130 | 137 | 128 | 138 | — | 687 | 691 | 783 | 795 | 1365 | 1332 | 1358 | 1299 |
| England and Wales | 126 | 122 | 121 | 136 | — | 629 | 637 | 678 | 712 | 1281 | 1287 | 1232 | 1282 |
| | | | | | *Ratio to rate at same ages in England and Wales taken as 100* | | | | | | | | |
| Northern | 95 | 85 | 102 | 96 | | 86 | 82 | 88 | 90 | 88 | 87 | 84 | 87 |
| East and West Ridings | 98 | 97 | 97 | — | — | 95 | 95 | 95 | — | 99 | 99 | 100 | — |
| North western | 101 | 95 | 95 | 93 | — | 94 | 95 | 89 | 95 | 95 | 92 | 96 | 93 |
| Wales | 94 | 89 | 103 | 95 | — | 98 | 97 | 94 | 94 | 91 | 92 | 98 | 93 |
| North midland and eastern | 104 | 101 | 97 | — | — | 99 | 105 | 97 | 96 | 94 | 97 | 102 | — |
| Midland | 93 | 106 | 107 | 104 | — | 102 | 101 | 104 | 113 | 110 | 109 | 103 | 101 |
| South western | 100 | 105 | 99 | 93 | — | 98 | 104 | 100 | 102 | 94 | 100 | 106 | 101 |
| South eastern and southern | 104 | 101 | 100 | 96 | — | 106 | 97 | 112 | 101 | 110 | 107 | 102 | 103 |
| Greater London | 104 | 112 | 106 | 101 | — | 109 | 108 | 116 | 112 | 105 | 105 | 110 | 101 |
| England and Wales | 100 | 100 | 100 | 100 | — | 100 | 100 | 100 | 100 | 100 | 100 | 100 | 100 |

Notes
(1) Northumberland, Cumberland, Durham, Westmorland, North Riding.
(2) Lancashire and Cheshire (with parts of Derbyshire).
(3) Shropshire, Hereford, Staffordshire, Warwick, Worcester.
(4) South-east (except Greater London) and southern region.
(5) Owing to changes in the standard regions some rates in 1964–67 were not available and others (in the south) are approximations. This applies also to 1921–30 for which the rates are taken from Registrar General's report for 1937 (Text volume, p. 187).

Looking first at the ratios at ages 25–44 relative to the national rate, indices below 100 are shown by Wales and the northern region until 1958, by the East and West Ridings of Yorkshire until 1963 and by the North West after 1954, whereas the south eastern, southern and London areas give ratios ranging from 101 to 112 until 1963 and the Midland (West) area showed indices of 104 to 107 after 1954.

At ages 45–64 low ratios between 82 and 96 in Wales and the northern regions have continued up to the present, averaging 8% below the national average, whereas in the two southern groups the average index has been 10% above the national level, with the midland and south-western regions intermediate. At 65 and over Wales and the northern areas showed ratios as much as 16% below, whilst those in the south averaged 5% above the national rate.

Whatever have been the factors responsible for producing lower breast cancer mortality in the northern part of England and in Wales than in the south at ages after 45, they have operated constantly since 1921 and there is no indication that they have changed appreciably. A curious feature of the trends of death rates at ages under 45 in Table I is the virtual disappearance of the southern excess after 1958. The average of the absolute rates per million in the southern and south-west parts of England in 1949–58 was 130 compared with 118 in the 3 northern regions and Wales; but in 1959–63 the averages were 123 and 122 respectively, and in 1964–67 they both rose, to 132 and 130. Such a closure of the north/south gap makes unlikely any hypothesis of a lower susceptibility due to different ethnic origins of the populations, at any rate for the rapidly growing breast cancers. As Fig. 1 shows, however, no such disappearance has occurred of the north/south difference at ages 45–64 although at later ages there has been a recent narrowing of the gap. Attempts to explain the excess of stomach cancer in Wales by a special Celtic susceptibility have proved ill-founded in the past, and there is no reason to believe that the regional differences in the incidence of breast cancer arise from differing ethnic origins of the populations.

*Relation to cancer of the uterus*

Another problem is why the national rates of dying from the slow growing types of breast cancer has risen steadily at ages 45–64 since 1950 whilst the gap between the Greater London rate and northern rates has increased as seen in Fig. 1 and Table I. A factor which may be important in this connection is the curious inverse relation between incidence of breast and uterine cancers in the regions, as shown in Table II. Such an inverse relationship in the social classes of England and Wales was pointed out in 1938 (Registrar General) but relations of the same kind in the different regions have not been examined in any detail. The ratio of breast cancer death rate to uterine rate in England and Wales at ages 45–64 has more than doubled since 1921–30, from 1·17 to 2·55 (due perhaps to improved survival of uterine cases), and it rose in every region except Wales during 1949–63. In 1954–58 the ratio was below 1·88 in each northern region and Wales but exceeded 2·35 in every other region except the Midland (2·1).

Despite this regional contrast in the ratio the aggregate death rate from breast and uterine cancers ranged only from 889 to 980 per million with no sign of a significant regional pattern. This was true also in 1949–53 and in the whole period 1949–58 the coefficient of variation of the aggregate rates in the nine regions was only 2·7%. Such a constancy of regional distribution is remarkable for a pair of causes separately classified in the international list. In 1957–67 the southern areas showed some excess over the north in the aggregate rate.

Table II shows how the death rates from uterine cancer (which consists mainly of cervix cancer) have changed in recent years. At ages 25–44 the secular changes have not been large, but in contrast with breast cancer the rates in the northern areas and Wales have been higher than in the south in each time period. At ages 45–64 the national death rate has fallen from 345 in 1949–53 to 279 in 1964–67, and the average death rate in 1949–63 was 380 in the northern region compared with 273 in Greater London.

In a study of comparative mortality ratios at all ages from cervix cancer in the 48 county boroughs with over 100,000 population in 1950–52 (Stocks, 1955*b*) it appeared that in the four cities of the northern region the C.M.R.'s were 197, 183, 175 and 162, in six cities of the East and West Ridings they were 175, 172, 162, 141, 114, 109; and in 12 cities of the North West region they were 135, 134, 131, 126, 119, 118, 117, 111, 110, 106, 99, 97 and in the three Welsh cities 152, 129 and

TABLE II.—*Death Rates from Cancer of the Uterus in the Regions of England and Wales. Ratios to Breast Cancer Rates and Totals of the Two Sites*

| Region* | Ages 25–44 1949–53 | 1954–58 | 1959–63 | 1964–67 | Ages 45–64 1921–30 | 1949–53 | 1954–58 | 1959–63 | 1964–67 | Ages 65 and over 1949–53 | 1954–58 | 1959–63 |
|---|---|---|---|---|---|---|---|---|---|---|---|---|
| | Death rates from cancer of uterus (per million) | | | | | | | | | | | |
| Northern | 80 | 77 | 83 | 67 | 708 | 442 | 377 | 321 | 324 | 614 | 717 | 539 |
| East and West Ridings | 60 | 60 | 80 | — | — | 430 | 354 | 297 | — | 698 | 601 | 596 |
| North-western | 69 | 78 | 78 | 69 | 564 | 371 | 324 | 303 | 311 | 635 | 602 | 566 |
| Wales | 76 | 75 | 75 | 99 | — | 393 | 352 | 351 | 358 | 605 | 569 | 548 |
| North midland and eastern | 60 | 64 | 63 | — | — | 310 | 275 | 273 | — | 595 | 523 | 529 |
| Midland | 75 | 60 | 67 | 67 | 509 | 326 | 335 | 278 | 287 | 606 | 557 | 523 |
| South-western | 55 | 62 | 73 | 68 | 538 | 344 | 286 | 280 | 357 | 583 | 534 | 514 |
| South-east and southern | 56 | 54 | 70 | 57 | 508 | 314 | 271 | 302 | 269 | 592 | 520 | 487 |
| Greater London | 46 | 51 | 57 | 50 | — | 305 | 264 | 250 | 218 | 568 | 500 | 555 |
| England and Wales | 55 | 60 | 71 | 60 | — | 348 | 302 | 288 | 279 | 610 | 559 | 538 |
| | Ratio of breast to uterine cancer rate | | | | | | | | | | | |
| Northern | 1·49 | 1·31 | 1·49 | 1·99 | 0·74 | 1·23 | 1·39 | 1·98 | 1·98 | 1·83 | 1·57 | 1·93 |
| East and West Ridings | 2·05 | 1·97 | 1·44 | — | — | 1·38 | 1·80 | 2·14 | — | 1·79 | 2·07 | 2·04 |
| North-western | 1·86 | 1·53 | 1·47 | 1·99 | 1·15 | 1·57 | 1·87 | 2·18 | 2·17 | 1·92 | 1·97 | 2·09 |
| Wales | 1·57 | 1·47 | 1·67 | 1·30 | — | 1·55 | 1·82 | 1·97 | 1·88 | 1·92 | 2·08 | 2·20 |
| North midland and eastern | 2·07 | 1·94 | 1·95 | — | — | 2·02 | 2·36 | 2·50 | — | 2·10 | 2·32 | 2·38 |
| Midland | 1·57 | 2·17 | 1·94 | 2·12 | 1·28 | 1·98 | 2·10 | 2·88 | 2·79 | 2·32 | 2·48 | 2·42 |
| South-western | 2·29 | 2·11 | 1·64 | 1·85 | 1·21 | 1·92 | 2·37 | 2·58 | 2·03 | 2·09 | 2·40 | 2·53 |
| South-east and southern | 2·32 | 2·30 | 1·73 | 2·33 | 1·41 | 2·12 | 2·47 | 2·38 | 2·68 | 2·39 | 2·37 | 2·67 |
| Greater London | 2·83 | 2·69 | 2·25 | 2·76 | — | 2·25 | 2·97 | 3·18 | 3·65 | 2·40 | 2·66 | 2·45 |
| England and Wales | 2·29 | 2·03 | 1·70 | 2·27 | (1·17) | 1·79 | 2·19 | 2·34 | 2·55 | 2·10 | 2·32 | 2·28 |
| | Total death rate for breast and uterine cancer (per million) | | | | | | | | | | | |
| Northern | 199 | 183 | 207 | 200 | — | 1026 | 985 | 926 | 998 | 1736 | 1842 | 1577 |
| East and West Ridings | 183 | 178 | 198 | — | — | 1024 | 958 | 933 | — | 1949 | 1846 | 1810 |
| North-western | 197 | 197 | 192 | 196 | — | 955 | 932 | 908 | 985 | 1855 | 1791 | 1750 |
| Wales | 195 | 186 | 200 | 228 | — | 1013 | 972 | 993 | 1030 | 1768 | 1753 | 1755 |
| North midland and eastern | 184 | 188 | 186 | — | — | 935 | 944 | 923 | — | 1843 | 1773 | 1776 |
| Midland | 193 | 190 | 197 | 209 | — | 970 | 980 | 981 | 1089 | 2010 | 1937 | 1791 |
| South-western | 181 | 193 | 193 | 194 | — | 961 | 947 | 957 | 1080 | 1793 | 1817 | 1814 |
| South-eastern and southern | 186 | 178 | 191 | 190 | — | 981 | 889 | 1059 | 989 | 2006 | 1755 | 1796 |
| Greater London | 176 | 188 | 185 | 188 | — | 1001 | 962 | 1085 | 1064 | 1933 | 1832 | 1913 |
| England and Wales | 181 | 182 | 192 | 196 | — | 977 | 939 | 966 | 991 | 1891 | 1846 | 1770 |

* See notes under Table I.

113. Only three of these 25 towns in the north and Wales had mortality ratios below 107, whereas 17 of the remaining 23 towns, in the south, east and midland regions, gave C.M.R.'s below 107 (the exceptions being Nottingham, Stoke-on-Trent, Coventry, and the seaports Plymouth, Portsmouth and Southampton). Some powerful factor enhancing mortality from cervix cancer was evidently operative in the north and Wales and in seaports generally.

FIG. 2.—Mean annual death rates in 1949–63 from cancers of the uterus and breast in the regions of England and Wales.

There was therefore up to 1963 a strong inverse relationship between mortality from breast and uterine cancers in the regions, as shown in Table II and Fig. 2, although the total rate for the two sites varied little. The northern excess in uterine cancer became less pronounced in 1959–63 but increased again in 1964–67. When looking for environmental factors to account for this regional pattern of uterine cancer it is noticeable that there is a strong resemblance to that for cardiovascular diseases, nephritis and bronchitis amongst women aged 25–54 in 1963–66. The rates for those diseases in the 15 Hospital Regions of England and Wales were analysed in connection with a geographical study of congenital malformations of the central nervous system (Stocks, 1970a) and the tabulation shows that the average death rates in the Newcastle, Manchester, Liverpool and Wales regions bear the following ratios to the average rate in the four Metropolitan regions comprising the south east of England. For chronic rheumatic heart 1·8, coronary disease 1·7, other heart diseases, vascular lesions of nervous system and nephritis 1·4, bronchitis 1·9, all other diseases 1·1. For uterine cancer at 45–64 in 1964–67 the ratio between the average rate in the northern, north west and Wales standard regions to that in Greater London was 1·6, and to that in the rest of the south-east and south it was 1·4. The explanation for the north-west/south-east distribution of cardiovascular diseases (illustrated by a map in the paper referred to) is not yet known but it is suspected that calcium or other constituents of water supplies may be responsible (Morris *et al.*, 1961), and if so this might account for the uterine cancer pattern also.

The remarkable constancy of the total death rates for breast and uterus at ages over 45 during 1949–58 despite the wide variations in the proportions of breast cancer in the regional totals suggests that there is a linkage between the susceptibilities to the two forms of cancer, that is that they may be controlled by the same gene. In that case a " choice " could be made as to which site is affected, depending upon the exogenous factors to which the individual was being exposed and upon the degree of weakening of the potentiality of the gene to maintain the resistance to the development of cancer through its control mechanism (Gedda and Brenci, 1969).

A method by which it might be discovered whether the capacity for resistance to breast and uterine cancers is controlled by the same gene or by different and independent genes would be by studies of the life histories of monozygotic and dizygotic twin pairs. Supposing the same gene to be responsible for cancer susceptibility in the two sites, the frequencies of occurrence of breast and uterine cancers in pairs of MZ twins would show a larger proportion of cases in which the breast was affected in one twin and the uterus in the other than could occur by chance. This would reduce the degree of concordance between breast and breast and between uterus and uterus in the pairs but would produce a degree of concordance between breast in one twin and uterus in the other much larger than has usually been found between different cancer sites. This would not occur if different genes controlled breast and uterus cancers.

Very large numbers of twin pairs would be needed for such a study, but registers of twins are being built up in a number of countries for purposes of such investigations, notably by the Gregor Mendel Institute of Medical Genetics and Twin Research in Rome. A simultaneous examination of the apparent linkage between gastric and intestinal cancers could be made from the same data (Stocks, 1970*b*).

Assuming that certain genes control cancer susceptibility in more than one part of the body and that the frequency of such genes in the population is constant through the country, the higher incidence of uterine cancer in the northern areas and Wales would on this hypothesis result from excess of some extraneous factor operating there, and the lower rates for breast cancer would be a secondary result of the genetic linkage with uterus. Alternatively there might be an excess of some extraneous factor acting on breast cancer incidence in the south-east of England with resulting lower incidence of uterine cancer secondary to the genetic linkage. A possible factor of the second kind is discussed in the next section.

*Dietary differences in the regions*

Breast cancer death rates in 22 countries during 1962–66 were related with the mean annual intake of fat, sugar, carbohydrates and meat per head of the population by Hems (1970) taking into account also the birth rates, parity and frequency of blood group A. Correlation coefficients between mortality and fat consumption were 0·675 at ages of death from 65 to 69 and 0·545 at ages 40–44, and with sugar the coefficients were 0·796 and 0·618 respectively. Breast cancer mortality appeared to be associated also with parity and blood group A, but at the later ages 74% of the total variation was accounted for by fat and sugar consumption. In view of this an examination has been made below of the relations between the rates of consumption of articles of diet in the regions of England and Wales and breast cancer death rates at three age periods.

Table III shows the mean weekly intake of the articles of food per head of the population in the years 1957 and 1962 in seven regions of England and in Greater London, Wales and Scotland, expressed in terms of the averages in England and Wales taken as 100. These are calculated from the data given in the annual reports of the National Food Survey Committee of the Ministry of Agriculture,

TABLE III.—*Indices of Consumption of Various Foods During* 1957 *and* 1962 *in the Regions of England and Wales, Compared with Breast Cancer Mortality at Three Age Periods in* 1954–63

| Region | Food intake weekly per head as percentage of average in England and Wales, in 1957, 1962 (mean annual) | | | | | | | Breast cancer in 1954–63% of national | | |
|---|---|---|---|---|---|---|---|---|---|---|
| | Liquid milk | Butter and cheese | Other fats | Fresh green veg. | Fresh fruit | Potatoes | Calcium content | Ages 25–44 | Ages 45–64 | Ages 65 up |
| Northern | 89* | 82* | 127* | 68* | 95* | 94* | 94 | 93 | 85 | 85 |
| East and West Ridings | 88* | 79* | 127* | 70* | 86* | 94* | 92* | 97 | 95 | 99 |
| North-western | 101 | 96 | 110 | 63 | 83 | 102 | 99 | 95 | 92 | 94 |
| North midland and eastern | 101 | 103 | 110 | 110 | 96 | 101 | 100* | 99 | 101 | 100 |
| Midland (west) | 102 | 100 | 95 | 110 | 90 | 110 | 103 | 106 | 102 | 106 |
| South-western | 102 | 115 | 91 | 128 | 92 | 108 | 103 | 102 | 102 | 103 |
| South eastern and southern† | 106 | 106 | 96 | 121 | 102 | 93 | 104 | 102 | 102 | 104 |
| Greater London | 107 | 106 | 83 | 114 | 127 | 97 | 102 | 100 | 112 | 108 |
| Wales | 90 | 104 | 85 | 100 | 99 | 107 | 99 | 97 | 95 | 95 |
| England and Wales | 100 | 100 | 100 | 100 | 100 | 100 | 100 | 100 | 100 | 100 |

* Year 1962 only (separate rates not available for 1957).
† Excluding London.

Fisheries and Food for the two years (Ministry of Agriculture, etc., 1959, 1964) In the right hand columns are shown the female death rates from breast cancer at three ages in 1954–63, also in terms of the national rate.

The regions with low breast cancer rates are (as was seen in Table I) the three northern regions and Wales where the relative mortality indices are below 100 at all age periods. Consumption of liquid milk is likewise below average in the Northern and Yorkshire (Ridings) region and in Wales (indices 89, 88, 90) and is highest in the south-east and Greater London (106, 107) where the breast cancer rates are also highest. Consumption of butter and cheese also is below average in the three northerly regions (with indices 82, 79, 96) corresponding with breast cancer levels. Other fats, in contrast, have very high intake levels in the north (127, 127, 110). Fresh green vegetables show very low consumption levels in the north (68, 70, 63) and high levels in the south and south-east. Fresh fruit consumption shows no important differences except in London, and for potatoes the west midlands, south-west and Wales have highest indices (107), with low levels in the north and south. Calcium content of the weekly food is low in the northern and Yorkshire regions (94, 92) but the indices for the other regions range only from 99 to 104.

Sugar intake, in year 1967, not shown in the table, was 91% of the national average in the northern region and London, 96 in Yorkshire and the south-east, 101 in the south-west, 103 in the north midlands, 109 in Wales, 115 in the north-west and 116 in the midland region, no resemblance to the distribution of breast cancer rates being apparent.

The notable feature of Table III is the positive association between intake of liquid milk, butter and cheese on the one hand and breast cancer mortality in the regions, and the negative association with other fats such as margarine.* This

* The mean weekly intake of butter in England and Wales in 1957, 1962 was 5·9 oz., cheese 3·1 oz., milk 4·9 pints and other fats 6·3 oz.

FIG. 3.—Mean weekly consumption of fats per person, correlated with breast cancer death rates in English regions and in Wales.

is shown graphically in Fig. 3. Such a statistical relationship does not in itself prove causation but the fact that a similar association appears between the national rates for all fats and breast cancer rates in 22 countries (Hems, 1970) as mentioned above makes it advisable to pay attention to this as a possible factor contributing to the peculiar regional variation in breast cancer incidence. Another notable feature of Table III is the strong positive relation between the regional intake of fresh green vegetables and breast cancer mortality which contrasts with some previous findings of negative relations with incidence of cancer of other sites (Stocks, 1933, 1957).

### REFERENCES

GEDDA, L. AND BRENCI, G.—(1969) *Acta Genet. Med. Gemellol., Rome*, **4**, 329.

GREENWOOD, M.—(1925) ‘ Étude statistique sur le cancer du sein et d'uterus ’. League of Nations Health Organisation, Ch. 333, Vol. 1, p. 36.

HEMS, G.—(1970) *Br. J. Cancer*, **24**, 226.
MINISTRY OF AGRICULTURE, FISHERIES AND FOOD—(1959, 1964) Annual Reports of the National Food Survey Committee for 1957, 1962. London (H.M. Stationery Office).
MORRIS, J. N., CRAWFORD, M. D. AND HEADY, J. A.—(1961) *Lancet*, i, 860.
PASCUA, M.—(1956) *Bull. Wld Hlth Org.*, **15**, 5.
REGISTRAR GENERAL—(1938) Census of 1931, Occupational Mortality, Supplement.
STOCKS, P.—(1933) *Ann. Eugen.*, **5**, 237.—(1936) *Rep. Br. Emp. Cancer Campn*, Supplement.—(1955*a*) *Schweiz. Z. Path. Bakt.*, **15**, 706.—(1955*b*) *Br. J. Cancer*, **9**, 487.—(1957) *Rep. Br. Emp. Cancer Campn*, Supplement.—(1970*a*) *Br. J. prev. soc. Med.*, **24**, 67.—(1970*b*) *Br. J. Cancer*, **24**, 215.
DE WAARD, F., DE LAIVE, J. W. J. AND BAANDERS-VAN HALEWIJN, E. A.—(1960) *Br. J. Cancer*, **14**, 437.